# Relationship Between Frequency Domain Indicators of Heart Rate Variability and Both Age and Duration of Illness in Patients with Headache: A Cross-Sectional Study

**DOI:** 10.3390/biomedicines13010021

**Published:** 2024-12-26

**Authors:** Jong-Ho Kim, Jong-Hee Sohn, Sung-Mi Hwang, Jae-Jun Lee, Young-Suk Kwon

**Affiliations:** 1 Department of Anesthesiology and Pain Medicine, Chuncheon Sacred Heart Hospital, College of Medicine, Hallym University, Chuncheon 24253, Republic of Korea; poik99@hallym.or.kr (J.-H.K.); h70sm@hallym.or.kr (S.-M.H.); iloveu59@hallym.or.kr (J.-J.L.); 2 Institute of New Frontier Research, College of Medicine, Hallym University, Chuncheon 24253, Republic of Korea; deepfoci@hallym.or.kr; 3 Big Data Center, Chuncheon Sacred Heart Hospital, College of Medicine, Hallym University, Chuncheon 24253, Republic of Korea; 4 Department of Neurology, Chuncheon Sacred Heart Hospital, College of Medicine, Hallym University, Chuncheon 24253, Republic of Korea

**Keywords:** headache, heart rate variability, frequency domain, age, duration

## Abstract

**Background/Objectives**: One of the most prevalent neurological conditions in the world, headaches impact a large number of people. Patients who experience headaches often have autonomic nervous system dysfunction, which can influence the onset and duration of headaches. Heart rate variability (HRV) serves as an indicator of the autonomic nervous system’s activity and balance. In this study, we looked at the frequency domain of HRV in relation to age and headache duration in patients who had headaches. **Methods**: This cross-sectional research is a secondary analysis that makes use of data gathered from previously registered study projects. They were adult males and females aged 19 to 80. HRV was recorded in three channels over the course of 20 min using a 256 Hz sampling interval. HRV frequency domain was utilized to analyze the relationship between HRV and headache duration as well as between HRV and age. **Results**: In a smooth curve through the scatterplot using the locally estimated scatterplot smoothing line, low frequency/high frequency (LF/HF) declined beyond around 60 years of age, while the total power (TP) decreased until about 50 years of age, after which there was no discernible change. The duration of the headache did not significantly correlate with LF/HF or TP. However, TP decreased with age in the multivariate linear regression model (coefficient [95% confidence interval]: −0.003 [−0.003–−0.002]). **Conclusions**: There may be associations between HRV indices and age, but these associations may not be linear.

## 1. Introduction

Headaches rank as one of the most common neurological disorders worldwide, affecting many people and having a prevalence rate of 48.9% of the general population. [1]. Headache not only reduces the quality of daily life but also causes economic costs related to decreased productivity [2,3]. In particular, migraine and tension-type headache (TTH) are the most common types of headache, causing serious discomfort to patients [1,4].

The autonomic nervous system (ANS) plays an important role in regulating various bodily functions such as cardiovascular, digestive, and respiratory homeostasis [5]. The balance of the ANS is essential for maintaining health, and it is regulated by the interaction of the sympathetic and parasympathetic nerves [6,7,8]. Migraine and TTH patients often show dysfunction of the ANS, which can affect the occurrence and persistence of headaches [9,10,11]. Heart rate variability (HRV) is widely used as one of the methods to evaluate the function of the ANS [12,13,14]. Many studies have shown that HRV is observed in patients with headache [15,16,17,18,19,20]. Hence, HRV may be a useful measure of autonomic dysfunction in chronic migraine patients [16].

HRV reflects the activity and balance of the ANS as an indicator of measuring HRV [21,22]. In assessing HRV, frequency domain indicators are a way to understand the activity of the ANS by analyzing the fluctuations in heart rate as a function of frequency. Frequency domain indicators are often calculated through power spectrum analysis, where different frequency bands are associated with different levels of ANS activity. In particular, HRV can be used to evaluate the activity of sympathetic and parasympathetic nerves through the power of low-frequency (LF) and high-frequency (HF) bands [23,24]. The HF band affects the heart rate by combining both the parasympathetic and direct breathing effects [25]. In other studies, the LF range combines the effects of the sympathetic and the baroreceptor reflex [26,27]. Some studies have reported the presence of a resonance response in HRV at frequencies close to 0.1 and 0.25 Hz [28]. Stimulation at a speed close to the resonance frequency can result in large amplitude blood pressure oscillations that can increase the sensitivity of the baroreceptor reflex over time [29,30,31]. Stimulation of the baroreceptor reflex by respiration and rhythmic skeletal muscle tension near the resonance frequency can cause an immediate large-scale increase in respiratory sinus arrhythmia compared to the resting baseline [29,32,33]. However, opinions are different on these issues, and the debate continues [28,29]. The LF/HF ratio is used as an important indicator representing the balance of the ANS, and it is associated with various health statuses [34]. The total power (TP) of frequency of HRV indicates the overall active status of the ANS, especially that of the sympathetic and parasympathetic nerves. High TP indicates good reactivity, controllability, and adaptability against various stimuli, such as stress and physical activity. Low TP presents functional decline of the ANS, and this outcome may be associated with chronic stress, fatigue, and disease [35,36].

Although there are many studies related to the association between HRV and the type of headache, research focusing on changes in the ANS with age and headache duration is relatively lacking to date. In this study, we investigated the LF/HF ratio and TP with age and headache duration in patients suffering from headaches, which provides an important insight into the overall activity and the balance of the ANS. With these tools, the diagnosis and treatment of headaches can be clarified and thereby contribute to the development of customized treatment strategies for personalized medicine.

## 2. Materials and Methods

### 2.1. Study Design

This study is a secondary analysis study using collected data from a previous research project registered in the Korean Clinical Trial Database. The information can be verified at the following URL: https://cris.nih.go.kr/cris/index/index.do (Study ID: KCT0008684; accessed on 22 October 2024). In the original project, the inclusion criteria encompassed adult men and women aged 19 to 80 seeking treatment for all types of headaches at the Chuncheon Sacred Heart Hospital in Chuncheon, South Korea, between 1 April 2023 and 22 February 2024, including both outpatients and inpatients, as well as emergency room patients. The causes of headaches included migraine, TTH, headache of vascular, infection, inflammation, or trauma origin (Table 1).

The diagnosis of headaches was classified according to the Korean Classification of Diseases (KCD) 8th edition [37]. The KCD is a system used in South Korea to classify diseases and causes of death systematically based on their similarities. It is utilized in medical record data, mortality statistics, and other health and demographic records. Based on the World Health Organization’s International Classification of Diseases [38], the KCD is adapted to suit the circumstances of South Korea, ensuring consistent and comparable data for health statistics.

Exclusion criteria were applied to individuals who declined participation, those unable to articulate their intentions due to mental or physical impairment, those incapable of completing surveys or responding to inquiries, and individuals younger than 19 or older than 80 years of age.

### 2.2. Ethical Approval and Informed Consent

This study was approved by the Chuncheon Sacred Heart Hospital Institutional Review Board (IRB) (No. 2024-08-003). All research activities strictly adhered to the ethical principles outlined in the Helsinki Declaration. Participants in the original study gave written informed consent, authorizing the use of their de-identified data for secondary analyses in future research projects that align with the general purpose of the original study. Based on this content, informed consent was deemed exempt from the IRB review.

### 2.3. Source and Processing of Data

With the exception of the HRV data, all the data used in this study were collected with a survey conducted in the previous study project. Age, sex, headache duration, and comorbidities were included. The duration of headache (days) was calculated by multiplying the number of months by 30 when expressed in months and by multiplying the number of years by 365 when expressed in years.

The comorbidities were the presence or absence of hypertension, diabetes, depression, and anxiety disorder through diagnosis from doctors.

#### 2.3.1. Heart Rate Variability

Collection and analysis of the HRV data were generally in accordance with the 1996 Task Force of the European Society of Cardiology and the North American Society of Pacing and Electrophysiology guidelines [35], but there were differences in some areas. The guidelines suggest that the collection of data should be measured in a stable environment and that the patient’s physical condition (rest state) should be kept constant, but this was not the case in our study. This study may be difficult to see in a completely stable state, as the secondary analysis study involved the task of collecting speech.

Electrocardiography (ECG) was recorded in a sitting position through three channels. Because a previous study included voice analysis of the subjects, they read predetermined sentences during the ECG recording. Although the ECG recording time was approximately 20 min, there were some differences in the effective measuring time depending on the subject’s speed of reading predetermined sentences (Table S1). The sampling interval of ECG to measure HRV was 256 Hz. Recording and sampling of ECG and measurement of HRV were conducted with HRV-Addon (LAXTHA, Daejeon, Republic of Korea).

#### 2.3.2. Data Processing

Because psychological factors such as adaptation to the environment, fatigue, and change in stress may occur in the initial phase and at the end of ECG collection, we removed the first 3 min and the last 3 min of HRV data. Since abnormal points caused by an artifact, physical movement, or error of the ECG measurement can occur, we assessed abnormal patterns of HRV using its original graphs and removed the affected parts. Additionally, if there was an abnormal pattern in one or more channels among the three channels in a specific section, we removed the HRV data from all three channels in that section.

The power spectrum density corresponding to a specific frequency band was calculated by integrating the processed HRV data using the Welch method. The method is a variant of the fast Fourier transform recommended by the guidelines [35], which divides the signal into segments and averages the spectrum of each segment, providing stability and smoothness of the spectrum [39]. The frequency was divided into three bands: very low frequency (VLF), LF, and HF, which included 0.0033–0.04, 0.04–0.15 Hz, and 0.15–0.4 Hz, respectively. The power of each frequency band for each patient was calculated using the trapezoidal integration method across the three channels. Then the mean of the three channels in VLF, LF, and HF was used as their representative readout. The LF/HF ratio and TP were calculated using the representative readout of VLF, LF, and HF. TP is the sum of VLF, LF, and HF.

### 2.4. Statistics

In the demographic data and the characteristics of HRV data, continuous data were expressed as a median and interquartile range and categorical data were expressed as a number and percentage. To analyze the association between HRV factors and age and between HRV factors and headache duration, we used a locally estimated scatterplot smoothing (LOESS) line to fit a smooth curve through the scatterplot. Visualizing the relationship of the HRV factors with age and headache duration as an optimal-fit curve makes it easier for readers to understand. Considering the covariates, we analyzed the unadjusted and adjusted association between LF/HF and age, LF/HF and headache duration, TP and age, and TP and headache duration using linear regression. The covariates included sex, type of headache (migraine, TTH, and others), hypertension, diabetes, depression, and anxiety. The first subgroup analysis was performed separately on patients with migraine and those with TTH. The second subgroup analysis was performed separately on patients aged <50 and those aged ≥50. Linear regression analysis is likely to cause overfitting when many variables are used, and the sample size is small. This causes the model to overfit the given data, learning the noise, such as random variability and unintentional variables in the data, and reducing the generalization performance. Therefore, we performed a multivariate analysis using only headache type, age, and headache duration as variables included in the linear regression analysis of the first subgroup.

Using Bonferroni correction, a *p*-value ≤ 0.0125 (0.05/4) was considered statistically significant. For all computational analyses and visual analyses, the Google Colab platform (Google LLC, Mountain View, CA, USA) was employed, leveraging its cloud-based Python (Python version 3.7) and R environments (R version 4.4.2).

## 3. Results

### 3.1. Characteristics of Patients and Their Heart Rate Variability

**Figure 1:**
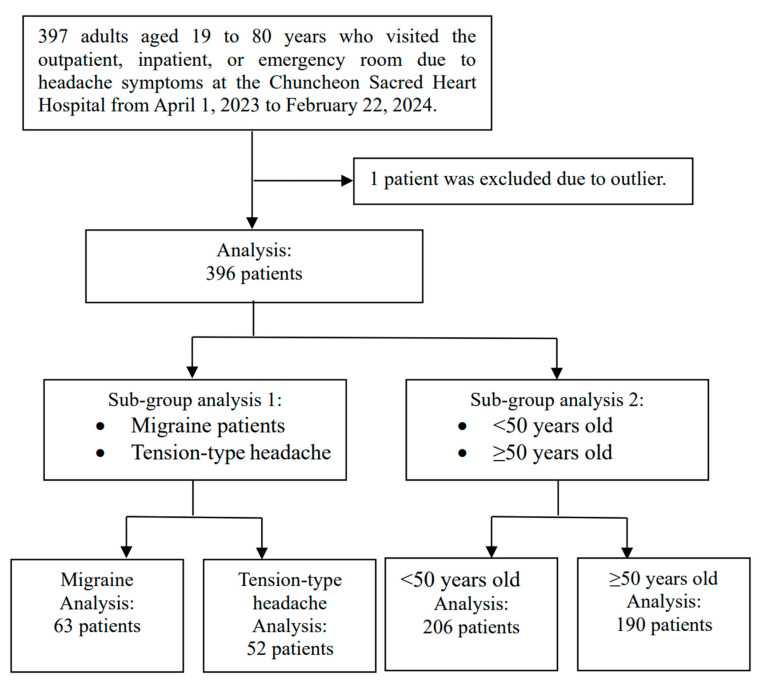
This flow chart illustrates the process of patient selection analysis for the study. The first step of the analysis includes all headache patients. The second step involves a subgroup analysis, which examines migraine and tension-type headache patients and further stratifies them based on whether their age is lower or higher than 50.

In the database of the original project, 397 patients with headaches were enrolled between 1 April 2023 and 22 February 2024. One patient with an outlier in headache duration was excluded, and 396 patients were analyzed. The study design, group, and subgroup formation principles are summarized in Figure 1. The characteristics of patients and HRV data are summarized in Table 2.

### 3.2. Association Between Age and the Heart Rate Variability Factors

**Figure 2:**
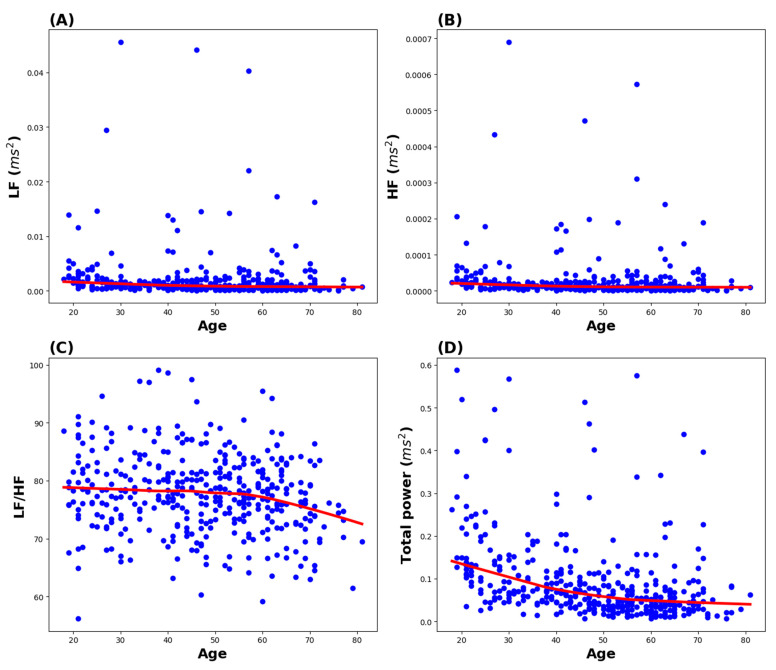
Association between age and the heart rate variability factors. Blue dots represent the scatterplot of age and the HRV factors (LF, HF, LF/HF, and TP). The red line is the LOESS line. (**A**) Association between age and LF, (**B**) association between age and HF, (**C**) association between age and the LF/HF ratio, (**D**) association between age and TP. HF, high frequency; LF, low frequency.

The associations between age and the HRV factors are summarized in Figure 2. In the LOESS lines of LF and HF, the changes with age did not show specific patterns. Although there were significant changes with age until 60 years in the LOESS lines of LF/HF, TP decreased from below 20 years to 50 years of age, and no significant change was observed after 50 years of age.

### 3.3. Association Between Headache Duration and the Heart Rate Variability Factors

**Figure 3:**
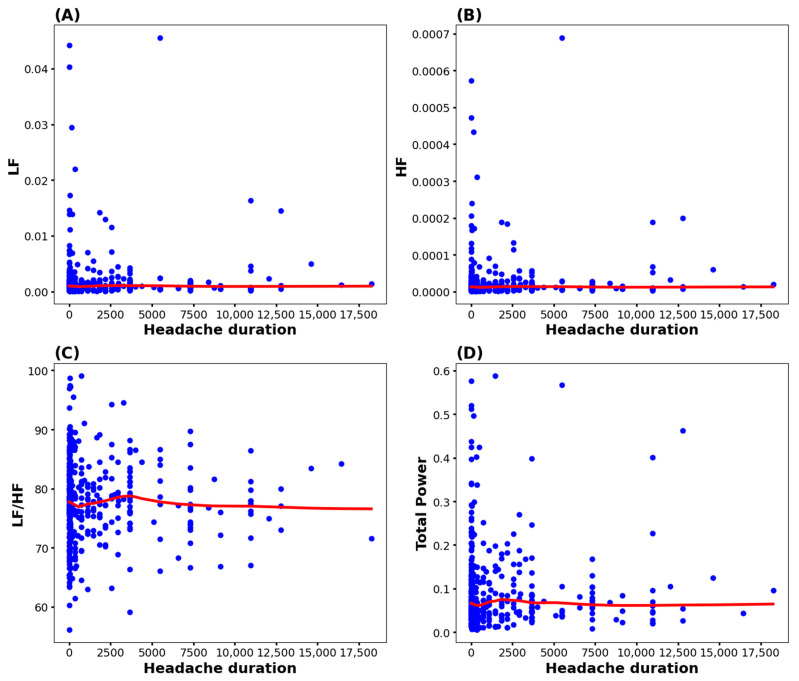
Association between the heart rate variability factors and headache duration. Blue dots represent the scatterplot of headache duration and the HRV factors (LF, HF, LF/HF, and TP). The red line is the LOESS line. (**A**) Association between headache duration and LF, (**B**) association between headache duration and HF, (**C**) association between headache duration and the LF/HF ratio, (**D**) association between headache duration and TP. HF, high frequency; LF, low frequency.

The associations between headache duration and the HRV factors are summarized in Figure 3. In all the LOESS lines of HRV factors, the changes in headache duration did not show specific patterns.

### 3.4. Association Between Age and Headache Duration in Relation to the Low/High-Frequency Ratio and the Total Power Through Linear Regression

The details of the association between age and headache duration to the LF/HF ratio and TP through linear regression are summarized in Table 3. In univariate analysis of the LF/HF ratio and TP, there were trends of decreasing the LF/HF ratio and TP with increasing age. In multivariable analysis, there was a trend of decreasing TP with increasing age. Details of the multivariable analysis are summarized in Table A1 (Appendix A).

The statistical power for analyzing the relationships between age, headache duration, the LF/HF ratio, and the TP using linear regression is summarized in Table S2. The statistical power for the LF/HF ratio is 0.999, and for TP, it is greater than 0.999.

### 3.5. The First Subgroup Analysis: Migraine and Tension-Type Headache

**Figure 4:**
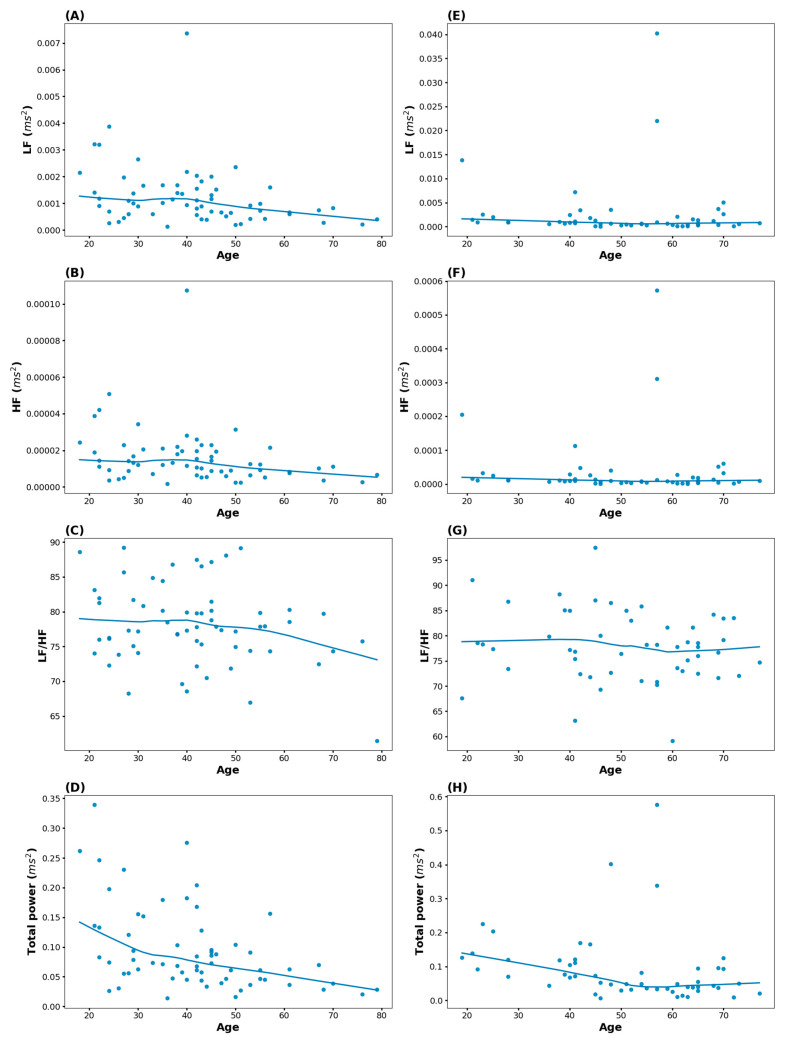
Association between HRV-related indices and age in migraine and tension-type headache (TTH). Blue dots are scatterplots of age and HRV-related indices. The blue line is the LOESS line. (**A**) Association between age and LF in migraine; (**B**) association between age and HF in migraine; (**C**) association between age and the LF/HF ratio in migraine; (**D**) association between age and TP in migraine; (**E**) association between age and LF in TTH; (**F**) association between age and HF in TTH; (**G**) association between age and the LF/HF ratio in TTH; (**H**) association between age and TP in TTH. HF, high frequency; HRV, heart rate variability; LF, low frequency.

Figure 4 depicts the association between HRV-related indices and age in migraine and TTH. In migraine, all HRV-related indices decreased overall as age increased. In TTH, TP decreased to the early fifties, with no significant change thereafter.

**Figure 5:**
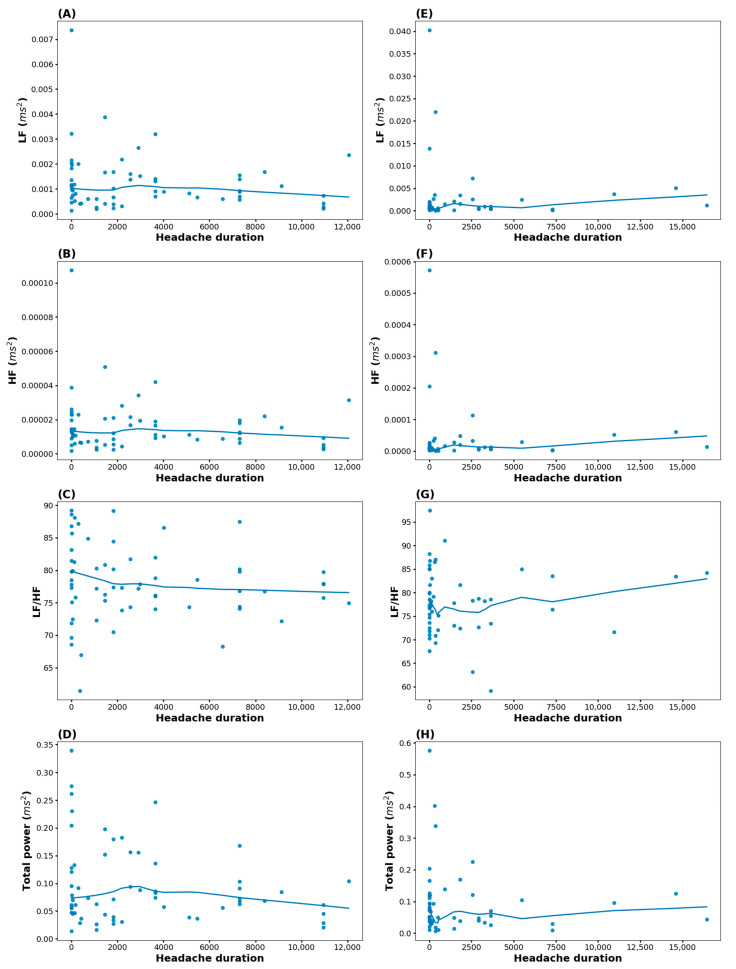
Association between HRV-related indices and headache duration in migraine and tension-type headache. Blue dots are scatterplots of headache duration and HRV-related indices. The blue line is the LOESS line. (**A**) Association between headache duration and LF in migraine; (**B**) association between headache duration and HF in migraine; (**C**) association between headache duration and the LF/HF ratio in migraine; (**D**) association between headache duration and TP in migraine; (**E**) association between headache duration and LF in TTH; (**F**) association between headache duration and HF in TTH; (**G**) association between headache duration and the LF/HF ratio in TTH; (**H**) association between headache duration and TP in TTH. HF, high frequency; HRV, heart rate variability; LF, low frequency.

Figure 5 depicts the association between HRV-related indices and headache duration in migraine and TTH. In migraine, there was no overall significant change as with increasing age. In TTH, the LF/HF ratio increased as age increased.

In migraine and TTH, the association between age and headache duration in relation to the LF/HF ratio and TP through linear regression is summarized in Table 4. Age had effects on TP in both migraine and TTH, but there was no significant effect on the LF/HF ratio. Headache duration did not have a significant effect on the LF/HF ratio and TP.

The statistical powers for analyzing the relationships between age and headache duration with the LF/HF ratio and TP through linear regression in patients with migraine and TTH are summarized in Table S3. In patients with migraine, the statistical power is 0.242 for the LF/HF ratio and 0.9 for TP. In patients with TTH, the statistical power is 0.067 for the LF/HF ratio and 0.17 for TP.

### 3.6. The Second Subgroup Analysis: <50 Years Old and ≥50 Years Old

Multivariate linear regression analysis showed that TP decreased with increasing age in headache patients younger than 50 years, and the LF/HF ratio decreased with increasing age in headache patients older than 50 years. The results of the linear regression analysis of the effects of age and headache duration on the LF/HF ratio and TP in headache patients under 50 years of age and headache patients over 50 years of age are summarized in Table 5. Details of the multivariate analysis are summarized in Table A2.

The statistical powers for analyzing the relationships between age and headache duration with the LF/HF ratio and TP through linear regression in patients who are <50 and ≥50 years old are summarized in Table S4. In patients who are <50 years old, the statistical power is 0.85 for the LF/HF ratio and 0.983 for TP. In patients with ≥50 years old, the statistical power is 0.665 for the LF/HF ratio and 0.711 for TP.

## 4. Discussion

This cross-sectional study investigated the relationship between HRV-related indices, especially the LF/HF ratio and TP, and both age and headache duration in 396 patients with headaches. In the scatterplot and the respective LOESS lines, the LF/HF ratio decreased after around 60 years of age, and TP decreased until around 50 years of age, while there were no significant changes beyond this age. There was no significant association between headache duration and both the LF/HF ratio and TP. However, since the multivariate linear regression model of all headache patients did not show significant results except for decreasing TP according to increasing age, subgroup analyses were performed before and after the age of 50. The results showed that TP significantly decreased with increasing age before the patients reached the age of 50, and the LF/HF ratio significantly decreased with increasing age after the age of 50. In the LOESS line of headache type with respect to age, there were different patterns between migraine and TTH. Migraine decreased the LF/HF ratio after around 50 years of age and decreased TP with increasing age. In TTH, TP decreased until around 50 years of age, with no subsequent significant change.

HRV reflects control of autonomic nerve systems affected by respiration, blood pressure, and brain and reflects activity of the sympathetic and parasympathetic nerve systems through measurement of HRV. For these reasons, many studies have used HRV to assess the ANS function [9,12,15,17,19,40,41,42]. Generally, the frequency domain indicators such as LF, HF, and TP of HRV decrease with increasing age in healthy Asian adults [40], and the LF/HF ratio is higher in old age than in young age [42]. However, in our results, the pattern of changes with age in patients suffering from headaches differed from that in healthy individuals. Differences in HRV between normal individuals and headache patients with increasing age suggest that ANS changes in headache patients with increasing age may differ from those in normal individuals.

The effect of HRV on headache sufferers has been reported in several studies [9,12,15,19,41,42]. However, there is limited research showing the frequency domain of HRV and its relationship to age. Tumurbaatar and colleagues investigated HRV in patients with TTH and found that there was no significant correlation between age and both LF/HF and TP [15]. Consistent with their study, no significant association was found between age and both the LF/HF ratio and TP in our analysis. However, Tumurbaatar et al. did not provide information on other types of headaches or details on the relationship between age and headache duration. Although Qavi and colleagues reported that LF/HF in TTH is higher than in healthy controls, they found no significant difference in migraine. Additionally, their results failed to show an association between frequency–domain indicators of HRV and both age and headache duration [41].

Research on the effect of headache duration on HRV is more limited. To our knowledge, no studies have investigated the relationship between headache duration and HRV. Patients with chronic pain have decreased HRV and baroreflex sensitivity due to altered cardiac activity of both sympathetic and parasympathetic nerves, which shifts the balance toward the prevalence of sympathetic tone associated with catecholamine release [43,44,45]. Although the relationship between pain and duration is unknown, it is possible that patients who have had headaches for a long period of time may have increased sympathetic tone, resulting in an increased LF/HF ratio or TP. However, these speculations are based on generalized pain, which may be different from headache, and our results did not show significant changes in the frequency domain of HRV in patients with headaches.

This study included patients with a variety of headache types and pathophysiologies. This is distinct from previous reports and may be the cause of differing results from previous studies in patients diagnosed with specific headaches. Accordingly, we also performed sub-analyses for the most common headaches, migraine, and TTH. In migraine, there is an overall decrease on the LOESS line, but decreased LF, TF, and TP are also seen in healthy people [40], so it may be difficult to attribute this feature to migraine. However, further studies may be needed after collecting samples because some results of migraine and TTH have insufficient statistical power in sub-group analysis. The LF/HF ratio is typically increased in older adults but can be decreased in cases of sympathetic nervous system inhibition or parasympathetic nervous system overactivity. In TTH, TP on the LOESS line decreased until age 50; there was no significant change thereafter, and the regression analysis results showed no significance. These results were similar to the results of all headache patients and were consistent with the results of previous studies. In the case of TTH, the effect of age may be limited. However, further research is needed to determine the exact causes.

The strength of this study is that it analyzed the association between HRV frequency domain and age and headache duration in headache patients and investigated changes in the respective subtypes. Nevertheless, this study has some limitations. First, because we only collected data at a point in time for each individual, we are not aware of how HRV indicators change over time and are not able to infer cause-and-effect relationships. Second, because we included only patients who visited the hospital due to headaches, there may be selection bias that excludes those who had headaches but who did not visit the hospital. Third, because it is difficult to investigate many variables simultaneously, it is difficult to control confounding variables. In particular, because the subtype of headache had small sample sizes, we could not conduct a multivariable analysis. Additionally, anxiety and depression are associated with HRV. We added the diagnosis of anxiety and depression as variables in the analysis, but we did not include the level of anxiety and depression when examining HRV. Therefore, future studies will also need to consider psychological parameters such as anxiety and the measurement of physiological symptoms of stress or other important symptom signs. It may also be important to evaluate the social support provided by friends or partners that may help people with headaches. Fourth, we did not control the respiratory rate during the experiment. Specifically, since data collection involved patients reading predetermined sentences, this may have further influenced their respiratory rate. As a result, the effects of cardiopulmonary interactions could have impacted HRV measurements and the interpretation of the results.

## 5. Conclusions

We investigated the association between frequency domain indices of HRV and both age and headache duration using data from 396 patients with headaches. Overall, there was no significant association between HRV indices and age or headache duration. However, considering the meaningful differences observed in the subgroup analysis, the relationship may be nonlinear. This possibility could inspire future studies focusing on ANS changes according to the age of headache patients.

## Figures and Tables

**Table 1:** Classification of diagnoses for migraine, tension-type headache (TTH), and other causes of headaches.

Type of Headache	Diagnosis
Migraine	migraine with or without aura, status migrainosus, complicated migraine, other and unspecified migraine.
TTH	acute, chronic, episodic, and unspecified TTH.
Other causes of headaches	other specified cerebrovascular diseases, other specified headache syndromes, dizziness and giddiness, benign intracranial hypertension, vascular headache, cluster headache syndrome, meningitis, meningitis in viral diseases, post-concussion syndrome, zoster without complication, herpes zoster, and unspecified headache.

**Table 2:** Characteristics and heart rate variability data in headache patients. Continuous data are expressed as a median (first quartile value, third quartile value), and categorical data are expressed as a number (%). Continuous data: age, power of the LF band, power of the HF band, LF/HF, and TP; categorical data: female, hypertension, diabetes, depression, anxiety. HF, high frequency; LF, low frequency.

	All Headache (n = 396)	Migraine (n = 63)	Tension-Type Headache (n = 52)
Age (year)	48 (35, 60)	42 (30, 50)	53 (41, 63)
Male	134 (33.8)	9 (14.3)	16 (28.9)
Female	262 (66.2)	54 (85.7)	37 (71.1)
Hypertension	82 (20.7)	6 (9.5)	10 (19.2)
Diabetes	44 (11.1)	2 (3.2)	6 (11.5)
Depression	47 (11.9)	13 (20.6)	8 (15.4)
Anxiety	16 (4.0)	2 (3.2)	2 (3.9)
Power of the LF band (ms^2^)	9.18 × 10^−4^ (4.69 × 10^−4^, 1.83 × 10^−3^)	9.31 × 10^−4^ (6.04 × 10^−4^, 1.58 × 10^−3^)	7.81 × 10^−4^ (4.32 × 10^−4^, 1.89 × 10^−3^)
Power of the HF band (ms^2^)	1.17 × 10^−5^ (6.29 × 10^−6^, 1.83 × 10^−5^)	1.20 × 10^−5^ (7.37 × 10^−6^, 2.01 × 10^−5^)	1.02 × 10^−5^ (6.14 × 10^−6^, 2.57 × 10^−5^)
LF/HF	77.8 (73.6, 81.6)	77.8 (74.7, 81.1)	77.8 (72.9, 83.1)
Total power (ms^2^)	0.065 (0.038, 0.113)	0.071 (0.046, 0.124)	0.054 (0.035, 0.113)

**Table 3:** Age and headache duration in relation to the LF/HF ratio and TP using univariable and multivariable linear regression analysis. CI, confidence interval; HF, high frequency; LF, low frequency.

		Univariable Analysis		Multivariable Analysis	
		Coefficient (95% CI)	*p*-Value	Coefficient (95% CI)	*p*-Value
LF/HF	Age	−0.07 (−0.11–−0.03)	0.001	−0.03 (−0.08–0.02)	0.284
	Headache duration	0 (0–0)	0.645	0 (0–0)	0.67
Total power	Age	−0.002 (−0.003–−0.002)	<0.001	−0.003 (−0.003–−0.002)	<0.001
	Headache duration	0 (0–0)	0.926	0 (0–0)	0.755

**Table 4:** Age and headache duration in relation to the LF/HF ratio and TP in migraine and tension-type headache using univariable and multivariable linear regression analysis. CI, confidence interval; HF, high frequency; LF, low frequency.

			Univariable Analysis		Multivariable Analysis	
			Coefficient (95% CI)	*p* Value	Coefficient (95% CI)	*p* Value
Migraine	LF/HF	Age	−0.103 (−0.203–−0.003)	0.043	−0.098 (−0.203–0.008)	0.069
		Headache duration	0 (−0.001–0)	0.355	0 (−0.001–0)	0.724
	Total power	Age	−0.002 (−0.003–−0.001)	<0.001	−0.002 (−0.003–−0.001)	<0.001
		Headache duration	0 (0–0)	0.120	0 (0–0)	0.592
Tension type headache	LF/HF	Age	−0.064 (−0.193–0.065)	0.325	−0.07 (−0.203–0.064)	0.299
		Headache duration	0 (0–0.001)	0.765	0 (0–0)	0.815
	Total power	Age	−0.002 (−0.004–0)	0.085	−0.002 (−0.004–0)	0.105
		Headache duration	0 (0–0)	0.498	0(0–0)	0.713

**Table 5:** Age and headache duration in relation to the LF/HF ratio and TP before and after 50 years of age using univariable and multivariable linear regression analysis. CI, confidence interval; HF, high frequency; LF, low frequency.

			Univariable Analysis		Multivariable Analysis	
			Coefficient (95% CI)	*p* Value	Coefficient (95% CI)	*p* Value
<50 years old	LF/HF	Age	−0.027 (−0.134–−0.079)	0.612	0.025 (−0.085–0.136)	0.654
		Headache duration	0 (−0.006–0)	0.042	0 (−0.001–0)	0.159
	Total power	Age	−0.004 (−0.006–−0.003)	<0.001	−0.004 (−0.006–−0.003)	<0.001
		Headache duration	0 (0–0)	0.622	0 (0–0)	0.128
≥50 years old	LF/HF	Age	−0.225 (−0.346–−0.103)	0.000	−0.184 (−0.309–−0.058)	0.004
		Headache duration	0 (0–0)	0.159	0 (0–0)	0.089
	Total power	Age	0 (−0.001–0.002)	0.831	0 (−0.002–0.001)	0.707
		Headache duration	0 (0–0)	0.893	0 (0–0)	0.958

## Data Availability

The datasets presented in this article are not readily available because the study is ongoing, and data collection is still in progress. Requests to access the datasets should be directed to the corresponding author’s email.

## Supplementary Materials

The following supporting information can be downloaded at https://www.mdpi.com/article/10.3390/biomedicines13010021/s1, Table S1: The original text (Korean) and the translation (English) of the sentences the subject read during the ECG data collection; Table S2: Statistical power for linear regression analysis of the LF/HF ratio and TP in patients with headache; Table S3: Statistical power for linear regression analysis of the LF/HF ratio and TP in patients with migraine and tension-type headache; Table S4: Statistical power for linear regression analysis of the LF/HF ratio and TP in patients who are <50 and ≥50 years old.

## Appendix A

**Table A1:** Results of multivariable linear regression in the LF/HF ratio and TP of heart rate variability. CI, confidence interval; DM, diabetes mellitus; HF, high frequency; HTN, hypertension; LF, low frequency.

		Coefficient (95% CI)	*p* Value
LF/HF	Male	2.03 (0.6–3.46)	0.005
	Migraine	0.21 (−1.69–2.11)	0.827
	Tension-type headache	0.46 (−1.49–2.41)	0.642
	Age	−0.03 (−0.08–0.02)	0.284
	Headache duration	0 (0–0)	0.67
	HTN	−0.87 (−2.57–0.83)	0.313
	DM	−4.11 (−6.3–−1.91)	<0.001
	Anxiety	0.62 (−2.75–4)	0.717
	Depression	−0.34 (−2.45–1.77)	0.75
Total power	Male	0 (−0.02–0.02)	0.988
	Migraine	−0.02(−0.04–0.01)	0.241
	Tension-type headache	0 (−0.02–0.03)	0.769
	Age	−0.003 (−0.003–−0.002)	<0.001
	Headache duration	0 (0–0)	0.755
	HTN	0.02 (−0.01–0.04)	0.123
	DM	0.02 (−0.01–0.05)	0.213
	Anxiety	0.02 (−0.03–0.07)	0.401
	Depression	0.02 (−0.01–0.05)	0.248

**Table A2:** Multivariable linear regression analysis of the effects of age and headache duration on the LF/HF ratio and TP in headache patients under 50 years of age and headache patients over 50 years of age. CI, confidence interval; DM, diabetes mellitus; HF, high frequency; HTN, hypertension; LF, low frequency.

			Coefficient (95% CI)	*p* Value
<50 years old	LF/HF	Male	3.436 (1.374–5.498)	0.001
		Migraine	1.609 (−0.823–4.04)	0.193
		Tension-type headache	1.374 (−1.752–4.501)	0.387
		Age	0.025 (−0.085–0.136)	0.654
		Headache duration	0 (−0.001–0)	0.159
		HTN	−1.181 (−4.512–2.151)	0.485
		DM	−10.3 (−18.459–−2.141)	0.014
		Anxiety	1.607 (−3.905–7.118)	0.566
		Depression	−0.297 (−3.896–3.301)	0.871
	Total power	Male	0.005 (−0.025–0.035)	0.757
		Migraine	−0.018 (−0.053–0.017)	0.31
		Tension-type headache	0.001 (−0.045–0.046)	0.971
		Age	−0.004 (−0.006–−0.003)	<0.001
		Headache duration	0 (0–0)	0.128
		HTN	0.017 (−0.032–0.065)	0.495
		DM	−0.06 (−0.179–0.058)	0.317
		Anxiety	−0.053 (−0.133–0.027)	0.191
		Depression	−0.006 (−0.058–0.046)	0.825
≥50 years old	LF/HF	Male	0.203 (−1.728–2.134)	0.836
		Migraine	−2.424 (−5.69–0.842)	0.145
		Tension-type headache	−0.346 (−2.768–2.075)	0.778
		Age	−0.184 (−0.309–−0.058)	0.004
		Headache duration	0 (0–0)	0.089
		HTN	−0.826 (−2.68–1.029)	0.381
		DM	−3.021 (−5.163–−0.879)	0.006
		Anxiety	0.284 (−3.825–4.394)	0.892
		Depression	−0.22 (−2.719–2.28)	0.863
	Total power	Male	−0.004 (−0.028–0.019)	0.725
		Migraine	−0.007 (−0.047–0.033)	0.72
		Tension-type headache	0.007 (−0.022–0.037)	0.637
		Age	0 (−0.002–0.001)	0.707
		Headache duration	0 (0–0)	0.958
		HTN	0.017 (−0.005–0.04)	0.132
		DM	0.02 (−0.007–0.046)	0.141
		Anxiety	0.067 (0.017–0.117)	0.009
		Depression	0.022 (−0.008–0.052)	0.155
