# Supplementary material for: Relationship Between Frequency Domain Indicators of Heart Rate Variability and Both Age and Duration of Illness in Patients with Headache: A Cross-Sectional Study
Source: Biomedicines. 2024 Dec 26;13(1):21. doi: 10.3390/biomedicines13010021 (PMC11762689; doi:10.3390/biomedicines13010021)
Supplement: Supplementary file 1 [file biomedicines-13-00021-s001.zip › biomedicines-3349158-supplementary.pdf]

Table S1. The original text (Korean) and the translation (English) of the sentences the subject read during the ECG data collection.

Original sentence	Translation sentence
바가지, 버섯, 보석, 부자	bowl, mushroom, jewel, rich
아들과 병원에 가보니 하필 늑막염이었다.	When I went to the hospital with my son, it turn ed out to be pleurisy.
애달픈 개처럼 해 질 녘까지 엄마를 기다렸다.	I waited for my mother until sunset like a sad d og.
어제는 허리가 아파서 거동이 불편했다.	Yesterday, my back hurt so much that I had diffi culty moving around.
에누리 없이 게와 고등어를 파는 사람들을 헤아려 보았다.	I counted the people who sold crabs and macker el without any discount.
오전에 호루라기를 챙겨서 고사장으로 갔다.	I took the whistle in the morning and went to th e examination hall.
우리는 후식으로 과자를 먹으며 구름을 보았다.	We watched the clouds while eating cookies for dessert.
으름장을 놓아서 그를 흐느끼게 만들었다.	I made him sob by letting go of the bottle.
이동할 때를 기다리며 히히덕거렸다.	He giggled while waiting for the time to move.
야생화 향기를 맡아보더니 고개를 약간 갸웃거렸다.	He smelled the scent of wild flowers and tilted h is head slightly.
얘는 얘기꾼이라 걔보다 얘기를 잘해.	He's a storyteller, so he tells stories better than h er.
여러가지 현악기가 결이 곱다.	Many stringed instruments have beautiful grain.
예금 상품의 혜택을 계산해보았다.	We calculated the benefits of deposit products.
와전된 과거의 일이 화를 불러왔다.	Distorted past events have caused trouble.

왜가리가 괘씸하게도 횃대를 부러뜨렸다.	The magpie broke the torch in an annoying way.
외삼촌은 금융업 분야의 회식을 괴로워했다.	My uncle was troubled by dinner parties in the f inancial industry.
요즘 교사들 사이에서 효도 관광이 유행이다.	These days, filial piety tourism is popular amon g teachers.
워낙 권력을 좋아해서 훨씬 더 회장 자리에 집착했다.	Because he loved power so much, he was even more obsessed with the position of chairman.
웨딩드레스에 대해 궤변을 늘어놓으며 훼방을 놓았다.	He caused trouble by spouting nonsense about wedding dresses.
위대한 귀농인을 만나기 위해서 휘파람을 불며 옷 입고 나갔다.	I got dressed and went out, whistling, to meet th e great farmer.
유도부가 휴가지에서 귤을 사 왔다.	The judo club bought some tangerines while on vacation.
의사는 축의금과 함께 희소식을 전했다.	The doctor delivered the good news along with a congratulatory gift.
가평의 고추밭에서 기적적으로 구출되었다.	Miraculously rescued from a pepper field in Ga pyeong.
까치는 꾸물거리다가 구멍 사이에 끼었다.	The magpie hesitated and got stuck between the holes.
나무에 널린 니트들이 누구의 것인지 궁금했다.	I wondered who the knits hanging on the tree b elonged to.
다리 아래 사는 두더지는 땅을 디딜 수 없었다.	The mole living under the bridge could not step on the ground.
따가운 자외선에 뚜껑이 검은 빛을 띠었다.	The lid turned black under the hot UV rays.
라면을 먹으면서 솜이불 위에서 루마니아 축구 리그를 보았다.	I watched the Romanian football league while e ating ramen and sleeping on a cotton blanket.

마침내 무서운 미로에서 빠져나왔다.	Finally got out of the scary maze.
바다에서 부드러운 바람을 맞으며 비빔밥을 먹었다.	I ate bibimbap while feeling the gentle sea breeze.
빠르게 뛰다가 뿌리에 걸려서 발목을 삐었다.	I was running fast and tripped on a root and sprained my ankle.
사다리 위에서 수다를 떨다 보니 시간이 다 되었다.	As we were chatting on the ladder, time passed.
싸다고 하는 씨감자로 죽을 쑤다 말았다.	I ended up making porridge with the cheap seed potatoes.
자전거 가게에서 주민 회의가 지금 진행 중이다.	A town hall meeting is currently underway at the bike shop.
짜게 끓인 찌개를 쭈그려 앉아서 먹었다.	I squatted down and ate the salty stew.
차선을 넘나들며 치열하게 추격전을 벌였다.	They were in a fierce chase, weaving across lanes.
카메라와 쿠키를 들고 있는 남자가 가장 키가 크다.	The man holding the camera and cookies is the tallest.
타조는 투명한 유리에서 칼날과 티끌을 발견했다.	The ostrich found a blade and dust in the transparent glass.
파란 눈과 하얀 피부 덕분에 첫인상이 푸근해보였다.	Her first impression was warm thanks to her blue eyes and white skin.
하늘이는 후미진 골목 끝에서 히죽거렸다.	Haneul was giggling at the end of a back alley.

Table S2. Statistical power for linear regression analysis of the LF/HF ratio and total power in patients with headache.

Variable	Sample Size	Residual Degrees of Freedom	Significance Level (α)	Cohen's f^2	Statistical Power
LF/HF ratio	396	10	0.125	0.224	0.999

Total power	396	10	0.125	0.228	>0.999

Statistical Power: The power of the statistical test to detect a relationship between variables. Cohen's f^2: A measure of effect size in linear regression (Small: $f^2 = 0.02$; Medium: $f^2 = 0.15$; Large $f^2 = 0.35$)

Table S3. Statistical power for linear regression analysis of the LF/HF ratio and total power in patients with migraine and tension-type headache.

	Variable	Sample Size	Residual Degrees of Freedom	Significance Level (α)	Cohen's f^2	Statistical Power
Migraine	LF/HF ratio	63	60	0.125	0.072	0.242
	Total power	63	60	0.125	0.287	0.9
Tension-type headache	LF/HF ratio	52	49	0.125	0.028	0.067
	Total power	52	49	0.125	0.063	0.17

Statistical Power: The power of the statistical test to detect a relationship between variables. Cohen's f^2: A measure of effect size in linear regression (Small: $f^2 = 0.02$; Medium: $f^2 = 0.15$; Large $f^2 = 0.35$)

Table S4. Statistical power for linear regression analysis of the LF/HF ratio and total power in patients with <50 and ≥50 years old.

	Variable	Sample Size	Residual Degrees of Freedom	Significance Level (α)	Cohen's f^2	Statistical Power
<50 years old	LF/HF ratio	206	196	0.125	0.116	0.85
	Total power	206	196	0.125	0.181	0.983
≥50 years old	LF/HF ratio	190	180	0.125	0.093	0.665
	Total power	190	180	0.125	0.063	0.711

Statistical Power: The power of the statistical test to detect a relationship between variables. Cohen's f^2: A measure of effect size in linear regression (Small: $f^2 = 0.02$; Medium: $f^2 = 0.15$; Large $f^2 = 0.35$)
